# Assessing the functional consequence of loss of function variants using electronic medical record and large-scale genomics consortium efforts

**DOI:** 10.3389/fgene.2014.00105

**Published:** 2014-04-29

**Authors:** Patrick Sleiman, Jonathan Bradfield, Frank Mentch, Berta Almoguera, John Connolly, Hakon Hakonarson

**Affiliations:** ^1^Center for Applied Genomics, Abramson Research Center, The Children’s Hospital of PhiladelphiaPhiladelphia, PA, USA; ^2^Department of Pediatrics, Perelman School of Medicine, University of PennsylvaniaPhiladelphia, PA, USA

**Keywords:** loss of function (LOF), imputation, PCSK9, eMERGE, biorepository

## Abstract

Estimates from large scale genome sequencing studies indicate that each human carries up to 20 genetic variants that are predicted to results in loss of function (LOF) of protein-coding genes. While some are known disease-causing variants or common, tolerated, LOFs in non-essential genes, the majority remain of unknown consequence. We explore the possibility of using imputed GWAS data from large biorepositories such as the electronic medical record and genomics (eMERGE) consortium to determine the effects of rare LOFs. Here, we show that two hypocholesterolemia-associated LOF mutations in the *PCSK9* gene can be accurately imputed into large-scale GWAS datasets which raises the possibility of assessing LOFs through genomics-linked medical records.

## INTRODUCTION

Complete loss of function (LOF) variants are defined as variants expected to correlate with complete LOF of affected transcripts; i.e., nonsense mutations, splice site mutations, and insertion/deletion (indel) variants that result in downstream premature stop codons, or larger deletions removing either the first exon or more than 50% of the protein-coding sequence of the affected transcript (MacArthur et al., 2012). Partial LOF variants reduce gene activity but do not ablate it completely.

Data from the 1000 genomes project (1KGP), a large scale human genome sequencing study of 1,092 individuals from 14 populations, constructed using a combination of low-coverage whole-genome and exome sequencing data indicates that on average individuals carry ~150 LOFs (Genomes Project Consortium et al., 2012). However, as detailed in **Table 1**, the majority of LOFs are common (>5%) and are distributed across a very small number (100–200) of genes. Genes containing common LOFs are strongly enriched for functional categories related to olfactory reception that are apparently unessential and do not result in any severe medical consequence. LOF enriched genes are typically depleted for genes implicated in protein-binding, transcriptional regulation, and anatomical development. Common LOFs are also enriched at the 3′ ends of genes as these mutations escape nonsense-mediated decay and are less subject to purifying natural selection. Finally, at the most highly conserved coding sites, more than 90% of stop-gain and splice-disrupting variants have a frequency below 0.5%. The population frequency of individual LOFs would therefore appear to correlate with their potential to adversely affect human health.

**Table 1:** Loss of function allele counts in 1,092 human genomes across three allele frequency bins.

	Allele frequency (%)		
Variant type	<0.5	0.5–5	>5
Stop-gain	3.9–10	5.3–19	24–28
Stop-loss	1.0–1.2	1.0–1.9	2.1–2.8
Indel frameshift	1.0–1.3	11–24	60–66
Splice site donor	1.7–3.6	2.4–7.2	2.6–5.2
Splice site acceptor	1.5–2.9	1.5–4.0	2.1–4.6

The 1KGP data indicates that each individual carries 10–20 LOF variants with a minor allele frequency (MAF) below 0.5% (**Table 1**). As these LOFs are under purifying selection they are less likely to be present in non-essential genes and at low conservation sites and therefore are likely to present pathological candidates.

The population frequency of rare variants differs considerably compared with common variation. Variants with frequencies above 10% were found in all of the populations studied in the 1KGP (Genomes Project Consortium et al., 2012), albeit with differences in MAF. Low-frequency variants in the 0.5–5% range were also largely shared between ancestral groups with only 17% of variants observed in a single ancestry group. For rare frequency variants with MAFs <0.5%, the majority (53%) were observed in a single population. Population stratification therefore represents a major confounder for rare variant analyzes which would ideally be controlled using principal component analysis from high-density GWAS arrays to select ancestrally matched cases and controls.

As a consequence of their rarity, LOFs will have largely been overlooked in GWAS studies which are best suited to the study of variants with minor alleles >3–5%. However, due to their rarity, very large, GWAS-type sample sets will be necessary to determine phenotypic association.

PCSK9 is expressed primarily in the liver, it is a secreted protein that acts by reducing the amount of low density lipoprotein receptor (LDLR) at the cell surface. Structurally, the PCSK9 protein product is composed a signal peptide, a prodomain, a catalytic domain, and a C-terminal domain. Cleavage of the prodomain is required for PCSK9 maturation and secretion. Cleaved PCSK9 is transported along the secretory pathway, which ultimately promotes LDLR degradation [for review see (Marais et al., 2012)]. One LOF missense mutation in PCSK9, Q152H, has been shown to impair cleavage and hence inhibit PCSK9 secretion (Mayne et al., 2011). The Q152H LOF mutation was shown to result in a 79% decrease in circulating PCSK9 and a 48% decrease in LDL-C in carriers compared with non-carriers (Mayne et al., 2011). The C679*X* mutation results in a processed, partially-folded protein that remains in the ER and is not secreted. As LDLR is degraded at the cell surface and endosomes, the C679*X* mutant has no activity toward the LDLR because of its inability to leave the ER and traffic to LDLR (Benjannet et al., 2006). R46L is also a LOF PCSK9 mutation, the R46L-PCSK9 undergoes near normal autocatalytic cleavage and is secreted, yet cells expressing the mutant displayed a 16% increase in of cell surface LDLR and a 35% increase in internalized LDL compared with WT-PCSK9, suggesting that R46L causes hypocholesterolemia through a decreased ability to degrade LDLR (Cameron et al., 2006).

Mutations in *PCSK9* were first identified in two French families with hypercholesterolemia that screened negative for mutations in both the LDLR and the apolipoprotein B (apoB) genes (Abifadel et al., 2003). The hypercholesterolemia *PCSK9* mutations were all missense variants that are thought to confer a gain of function as overexpression of *pcsk9* in the liver of mice produces hypercholesterolemia by reducing LDLR numbers (Lambert et al., 2006).

In 2005, causative LOF mutations in *PCSK9* were identified in individuals with low plasma LDL-C levels, the LOF variants were shown to be present in ~2% of the African–American population but rare in European Americans (<0.1%; Cohen et al., 2005). LOF mutation carriers displayed reduced or no PCSK9 activity, and their plasma LDL-C levels were reduced by 40% compared with non-carriers. Further, coronary heart disease risk in those individuals was reduced by 88% compared to non-carriers (Cohen et al., 2006). This observation sparked interest in the biology of PCSK9 and led to the development of several LDL-reducing drugs (Stein et al., 2012).

While the cost of whole genome and exome sequencing experiments has dropped dramatically with improvements in yield from second generation sequencing technologies, very large scale studies remain prohibitively expensive. For sample sets with existing genotypes from dense whole-genome arrays, genotype imputation presents a viable alternative to direct sequencing. Data generated from large sequencing projects such as the 1KGP (Genomes Project Consortium et al., 2012) and the NHLBI exome sequencing project (ESP; Tennessen et al., 2012) is phased (Delaneau et al., 2012) and the haplotypes can be used as reference panel to impute missing variation into the sample genotype data (Howie et al., 2009). Recent improvements in imputation algorithms and the expansion of reference datasets have improved accuracy of imputation for even low MAF variants. Imputed data can then be annotated using tools developed for the annotation of sequencing data such as SnpEff (Cingolani et al., 2012) which determine the genomic location (i.e., exonic, intronic or intergenic, and the effects of variants, missense, nonsense etc. on known genes). Imputed LOF variants can then be assessed against binary phenotypes or quantitative laboratory values derived from patients electronic medical records (EMR).

We sought to determine if two PCSK9 LOF mutations that are present in the 1KGP data, the C679X nonsense mutation and the R46L missense mutation, could be imputed into our dataset and the previously reported association of the LOFs with decreased serum LDL-C replicated.

## MATERIALS AND METHODS

The Center for Applied Genomics (CAG) at The Children’s Hospital of Philadelphia (CHOP) maintains a biorepository of over 160,000 genotyped samples, 60,000 of which are pediatric samples randomly recruited from CHOP with complete EMRs. As a proof of principle, we imputed the proprotein convertase subtilisin kexin type 9 (*PCSK9*; NM_174936) LOFs C679X (dbSNP:rs28362286) and R46L (dbSNP:rs11591147) into a random selection of 8,028 unrelated samples of Northern European ancestry genotyped on the Illumina HumanHap 550 array from the CAG biorepository. The study was approved by the Institutional Review Board at the CHOP, and written informed consent for sample collection and DNA genotyping/sequencing was provided by the parents of all participating children.

Genetic ancestry was determined by computing principal components on the dataset using smartpca, a part of the EIGENSTRAT package, on 100,000 random autosomal SNPs in linkage equilibrium. Samples were clustered into 4 Continental ancestry groups (Caucasian, African including admixed African–American, Asian, and native American/admixed Hispanic) by K-means clustering using the kmeans package in R. The European ancestry grouping in our dataset mapped most closely to the HapMap CEU population of Utah residents with Northern and Western European ancestry from the CEPH collection^1^.

Duplicate samples and cryptic relatedness were assessed by pairwise IBD. IBD values were generated for all 8,028 samples of Northern European ancestry using the plink genome command. A random sample from any pair with a PI_HAT value exceeding 0.3 was excluded from further analysis.

Imputation of untyped markers (~39 M) was carried out using IMPUTE2 after prephasing with SHAPEIT. Each chromosome was prephased separately. Reference phased cosmopolitan haplotypes and recombination rates were obtained from the 1000 genomes project (1000 Genomes Phase I integrated variant set b37 March 2012 release). Imputation was carried out in 5Mb intervals using an effective population size of 20000 as recommended. As a measure of the overall imputation accuracy we compared the concordance between the imputed and known genotypes in the subset of SNPs for which genotyping data was available. At a call threshold of 0.9, over 99% of the imputed genotypes were called and over 96% of those were concordant with the known genotypes.

## RESULTS

Following imputation using SHAPEIT^2^ and IMPUTE2^3^ and annotation using SnpEff^4^, we extracted and additively re-encoded genotypes for C679X and R46L from the 8,028 European American samples from the CAG biorepository. Both variants were imputed with high confidence, info scores C679X = 0.9 and R46L = 1. The C679X mutation was previously reported to be present in 0.1% of European Americans (Cohen et al., 2005). We identified nine C679X carriers out of 8,028 samples for a frequency of 0.11%, consistent with previous reports. As the samples were randomly selected from the biorepository, not all contained serum lipid data in their EMR. Three of the nine C679X carriers had serum LDL data. The frequency of the R46L was also consistent with the NHLBI ESP data, homozygous wild-type R46L 0.98 (1432 unique individuals with lab values mean age 12.1 years); heterozygous R46L 0.02 (10 unique individuals with lab values mean age 13.5) and homozygous derived allele R46L 0.001 (12 unique individuals with lab values mean age 11.5). A total of twenty-two R46L carriers had LDL data in the EMR.

**FIGURE 1:**
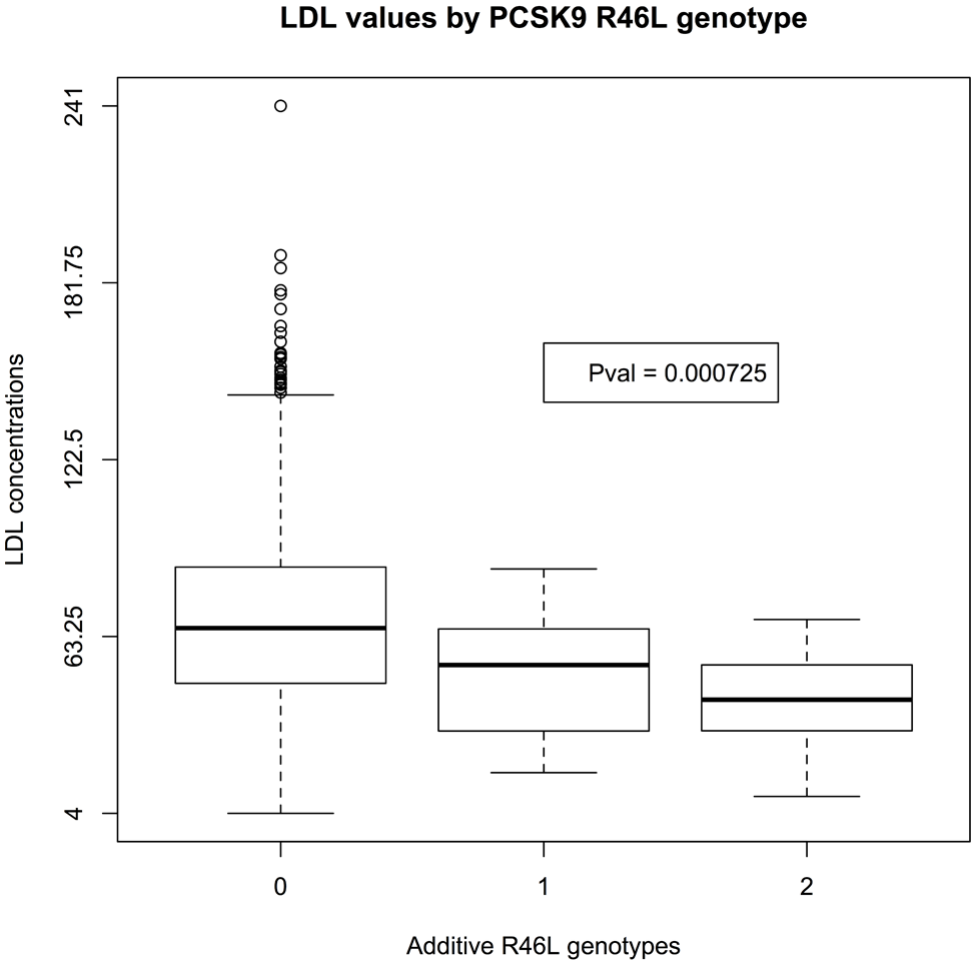
**Box and whisker plot of additively encoded *PCSK9* R46L genotypes on the X-axis versus age corrected serum LDL concentrations on the Y-axis (*n* = 1432; min LDL concentration 4; max LDL concentration 241; mean LDL concentration 85).** Genotype 0 indicates no copies of the mutant allele, genotype 1 indicates one copy of the mutant allele and genotype 2 indicates 2 copies of the mutant allele.

**FIGURE 2:**
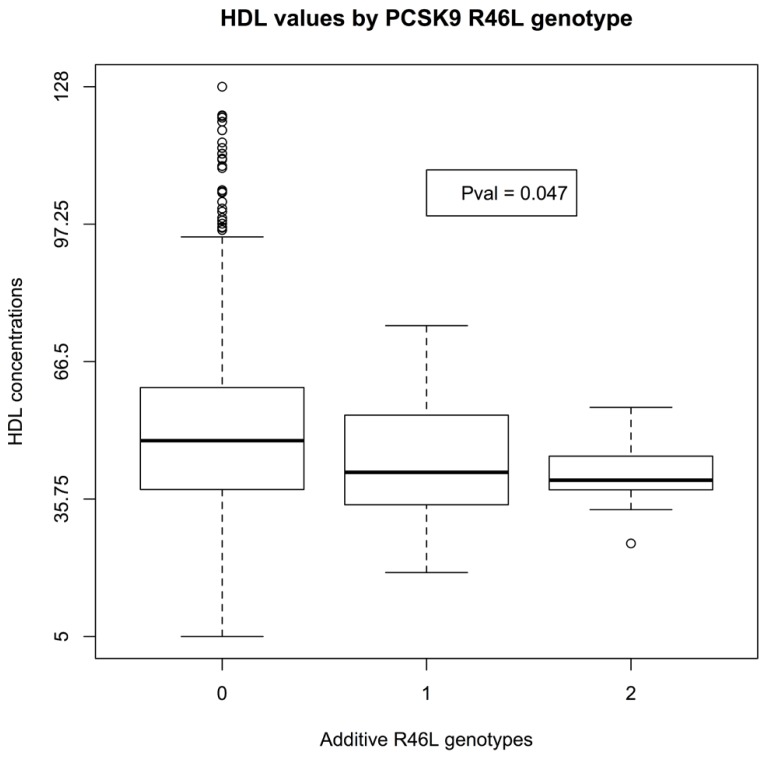
**Box and whisker plot of additively encoded *PCSK9* R46L genotypes on the X-axis versus age corrected serum HDL concentrations on the Y-axis (*n* = 1495; min HDL concentration 5; max HDL concentration 128; mean HDL concentration 47).** Genotype 0 indicates no copies of the mutant allele, genotype 1 indicates one copy of the mutant allele and genotype 2 indicates 2 copies of the mutant allele.

**FIGURE 3:**
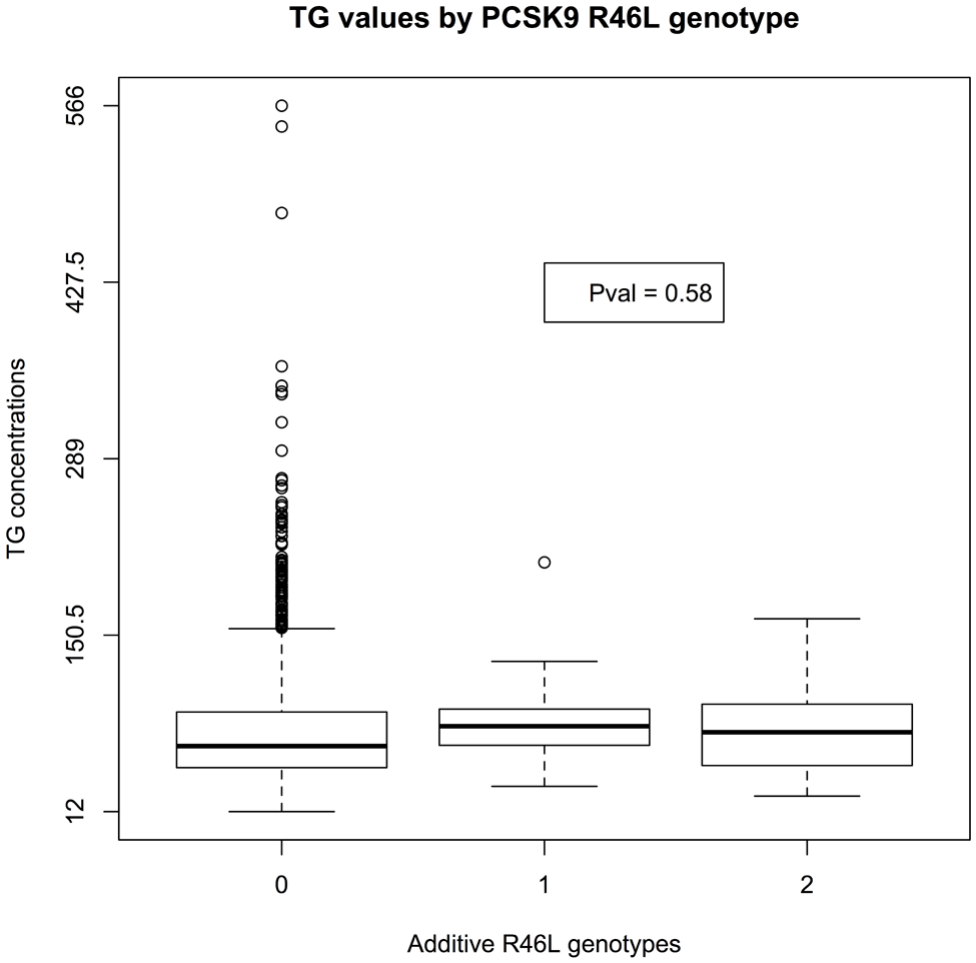
**Box and whisker plot of additively encoded *PCSK9* R46L genotypes on the X-axis versus age corrected serum triglyceride concentrations on the Y-axis (*n* = 1856; min TG concentration 12; max TG concentration 566; mean TG concentration 82).** Genotype 0 indicates no copies of the mutant allele, genotype 1 indicates one copy of the mutant allele and genotype 2 indicates 2 copies of the mutant allele.

There was insufficient data to assess the statistical significance of C679X genotypes. Linear regression of EMR-derived age-corrected serum LDL concentrations against R46L genotypes was statistically significant (*P*-value 7 × 10^-^^4^) and directions of effect consistent with the LOF allele reducing LDL cholesterol (**Figure 1**). Serum HDL concentrations also showed a trend toward association (*P*-value 0.04; **Figure 2**). By contrast, serum triglyceride levels showed no association with R46L genotype (*P*-value 0.58; **Figure 3**) as previously described (Kotowski et al., 2006). The mean age-adjusted LDL concentration for R46 wild-type homozygotes was 85.7, mean age-adjusted LDL concentration for R46L heterozygotes was 63 and 62.6 for R46L homozygotes which corresponds approximately to a 26% decrease of serum LDL consistent with the 23.5 mean LDL-C difference previously reported in European American R46L carriers (Kotowski et al., 2006).

## DISCUSSION

Recent genome sequencing studies have shown that each individual carries a significant number of variants that are predicted to result in a loss of protein function. The phenotypic effect of the majority of these LOFs remains to be determined. Here, we have shown a successful proof of concept that rare LOFs can be imputed into high density genotyping array data using data from large scale sequencing projects such as the 1KGP as a reference. While second generation sequencing remains prohibitively expensive in large numbers, high density genotyping data has been generated on hundreds of thousands of individuals. The eMERGE consortium biorepository includes ~60,000 individuals that have been genotyped on high-density GWA arrays (review at http://www.genome.gov/27540473), all of which has been linked with EMRs. As such eMERGE would be ideally suited for the assessment of rare LOF variants across multiple phenotypes either by direct assessment through single variant tests or through burden tests. For future analyses, in order to identify all possible association signals, the data would be analyzed using more than one statistical approach as detailed below.

Annotated, imputed variants, in vcf format^5^, would be analyzed for association using both single point and agglomerative tests. Single variant tests for association against the EMR traits would be implemented in EMMAX (Kang et al., 2010), a mixed model algorithm that controls for both population substructure and relatedness between individuals in the test. In addition to the principal components for population stratification applicable covariates such as age could be included. For the agglomerative gene-based association tests, three complementary algorithms, the sequence kernel association test (SKAT; Ionita-Laza et al., 2013), the variable threshold test (Price et al., 2010) and the combined multivariate and collapsing (CMC) test which assess the burden of variation within the gene (Li and Leal, 2008) would be implemented. Gene-based association tests can achieve substantial increases in power to detect associations with rare variation compared with single variant tests (Ionita-Laza et al., 2013).

We anticipate that for the single variant tests greatest power would be achieved against quantitative phenotypes such as lab values, however, gene burden scores could equally be applied using a pheWAS approach (Denny et al., 2010), i.e., EMR derived ICD9-based pseudo-case control analyzes for binary traits. These approaches will be validated on multiple LOF variants across the eMERGE networks in the near-future.

## Conflict of Interest Statement

The authors declare that the research was conducted in the absence of any commercial or financial relationships that could be construed as a potential conflict of interest.

## Footnotes

1 http://hapmap.ncbi.nlm.nih.gov

2 http://www.shapeit.fr

3 http://mathgen.stats.ox.ac.uk/impute/impute_v2.html

4 http://snpeff.sourceforge.net

5 http://www.1000genomes.org/wiki/analysis/variant-call-format/vcf-variant-call-format-version-42">
